# Studies on the antiviral activity of chebulinic acid against dengue and chikungunya viruses and in silico investigation of its mechanism of inhibition

**DOI:** 10.1038/s41598-022-13923-6

**Published:** 2022-06-21

**Authors:** Naiju Thomas, Poonam Patil, Anjana Sharma, Sandeep Kumar, Vikas Kumar Singh, Kalichamy Alagarasu, Deepti Parashar, Suman Tapryal

**Affiliations:** 1 Department of Biotechnology, School of Life Sciences, Central University of Rajasthan, NH-8, Bandarsindri, Ajmer, Rajasthan 305817 India; 2 Dengue and Chikungunya Group, ICMR-National Institute of Virology, 20-A Dr. Ambedkar Road, Pune, Maharashtra 411001 India

**Keywords:** Computational biology and bioinformatics, Drug discovery

## Abstract

Chebulinic acid (CA), originally isolated from the flower extract of the plant *Terminalia chebula*, has been shown to inhibit infection of herpes simplex virus-2 (HSV-2), suggestively by inhibiting the host entry step of viral infection. Like HSV-2, the dengue virus (DENV) and chikungunya virus (CHIKV) also use receptor glycosaminoglycans (GAG) to gain host entry, therefore, the activity of CA was tested against these viruses. Co-treatment of 8 µM CA with DENV-2 caused 2 log decrease in the virus titer (4.0 log_10_FFU/mL) at 120 h post infection, compared to virus control (5.95 log_10_FFU/mL). In contrast, no inhibitory effect of CA was observed against CHIKV infection under any condition. The mechanism of action of CA was investigated in silico by employing DENV-2 and CHIKV envelope glycoproteins. During docking, CA demonstrated equivalent binding at multiple sites on DENV-2 envelope protein, including GAG binding site, which have previously been reported to play a crucial role in host attachment and fusion, indicating blocking of these sites. However, CA did not show binding to the GAG binding site on envelope protein-2 of CHIKV. The in vitro and in silico findings suggest that CA possesses the ability to inhibit DENV-2 infection at the entry stage of its infection cycle and may be developed as a potential therapeutic agent against it.

## Introduction

The re-emerging arbovirus infections such as chikungunya and dengue have a devastating effect on human lives. These infections are transmitted to humans by mosquitoes of genus *Aedes* (*A. albopictus* and *A. aegypti*)^1,2^. The dengue and chikungunya viruses have caused a wave of large-scale epidemics gripping more than 100 countries from Europe, America, and Asia, with the number of new infections estimated to be in millions every year^3,4^. Both diseases show many common symptoms like chills, nausea, fever, headache, rash, myalgia, and even arthralgia. However, the dengue infection may lead to mild to severe hemorrhagic manifestations, accompanied by liver damage, thrombocytopenia, and even death. On the other hand, the chikungunya infection may have a lasting impact on the patient's life due to recurring polyarthralgia, leading to socio-economic loss^5^. Presently, specific antiviral drugs are not available for these diseases.

The dengue virus (DENV) is a single-stranded, positive-sense RNA virus in which the major envelope protein (E protein) and the pre membrane anchor protein (prM) form the outer shell of the virus^6^. E protein, a class II viral fusion protein, is essential for host receptor binding^7^ and fusion^8,9^. The E protein subunit consists of three domains, domain-I (EDI), EDII, and EDIII, and is found in a homodimeric state on the viral surface. The DENV may enter host cells through multiple receptors, such as glycosaminoglycans (heparan sulfate and lectins)^10^, Dendritic Cell-Specific Intercellular adhesion molecule-3-Grabbing Non-integrin (DC-SIGN)^11^, mannose receptor of macrophages^12^, lipopolysaccharide receptor CD14^10^, heat-shock proteins 70 and 90^10^, and ER chaperonin GRP78^13^. Recently, another type of carbohydrate molecule, neolactotetraosylceramide (nLc4Cer), a glycosphingolipid, has also been reported to contribute to DENV attachment and to serve as a possible co-receptor on the host cells^14^, indicating that DENV may enter different cell types through multiple receptors.

On the other hand, the chikungunya virus (CHIKV) is also a single-stranded, positive-sense RNA virus with a genome size of ~ 11.8 kb. It is covered by a lipid bilayer envelope^15^, containing the structural glycoproteins E1 and E2 with accessory peptides (E3 and 6 k)^16^ that assist in host cell entry. The latter facilitates cell attachment, whereas the former acts as a class II viral fusion protein^15,16^. Additionally, it has also been suggested that the A and B domains of E2 accommodate putative receptor binding sites^17^. Similar to DENV, CHIKV uses multiple receptors present on different types of host cells to gain host entry^18^; glycosaminoglycans (GAGs)^19^, prohibitin-1^20^, T-cell immunoglobulin mucin domain (TIM1), a phosphatidylserine receptor^21^, matrix remodelling associated protein (MXRA8)^22^ and ATP synthase β-subunit (ATPS-β, in insect cells)^23^ have all been known to assist host entry. GAGs, nLc4Cer, and prohibitin-1 are expressed on Vero cells^24^ and have hence been used in the current study for virus inhibition assays.

Chebulinic acid was originally isolated from the flower extract of the plant *Terminalia chebula*, a medicinal plant that belongs to the *Combretaceae* family and is one of the most documented plants in the ancient Indian Ayurvedic system of medicine^25,26^. Fruit and flower extracts have shown broad inhibitory activity against viruses such as swine influenza A virus^27^, Hepatitis B^28^, Hepatitis C^29^, HSV-1^30^, HSV-2^31^, HIV^32^, and cytomegalovirus^33^. *T. chebula* flower extract and purified tannins, e.g., chebulinic acid and chebulagic acid, have also been shown to inhibit HSV-2 infection in Vero cells, suggestively by blocking the virus entry into the host cells^31^; however, the mechanism of their action has not been investigated in depth. Glycosaminoglycans (GAGs) such as heparan sulfate (HS) are expressed on the surface of most mammalian cell types, and are used by various viruses to make first contact with the host cells, as observed in the case of HSV-2^34^, DENV^35^ and CHIKV^36^. Therefore, understanding the mechanism of action of CA is of huge significance for comprehending the full scope of its inhibition; furthermore it may also open up new avenues in the investigation of broad-range inhibitors against other viruses. In the current study, in vitro virus inhibition assays were performed to establish the antiviral activity of CA against DENV-2 and CHIKV. Subsequently, the mechanism of its action was investigated by in silico docking experiments using viral proteins, CA and receptor proteins. To our knowledge, this is the first such report of anti DENV activity of chebulinic acid and its source plant.

## Results

### Assessment of the cytotoxic effect of CA on Vero E6 cells

**Figure 1:**
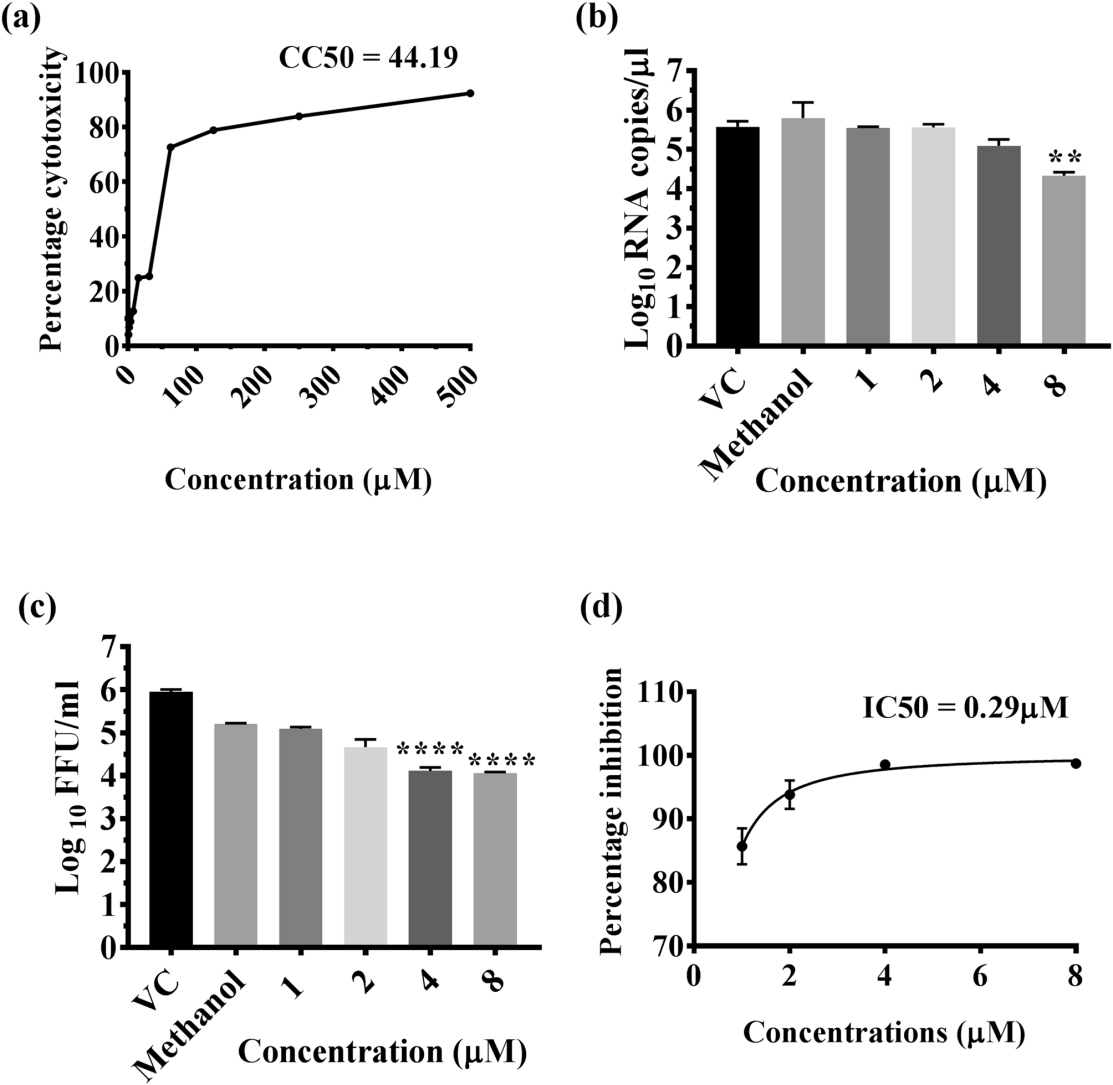
(**a**) Effect of Chebulinic acid (CA) on Vero E6 cells and dengue virus (DENV). (**a**) Cytotoxic effect of CA at various concentrations on Vero E6 cells. (**b**, **d**) Direct virucidal effect of CA at various concentrations on dengue virus infection at MOI = 0.1 in Vero E6 cells. Virus titer was assessed at 120 h post infection and expressed in terms of (**b**) Log_10_ viral RNA copies/µL and (**c**) Log_10_ focus forming units/ml, ***P value < 0.0001, *P < 0.05. (**d**) Percentage inhibition of DENV focus forming units/mL by CA compared to virus control (VC). All the experiments were performed in triplicates, and the data values are expressed as mean ± SEM.

The cytotoxicity of chebulinic acid was assessed in Vero E6 cells used in the assay. Concentrations ranging from 500 to 1 µM of the compound were tested for cytotoxicity. The results revealed that concentrations greater than 15 µM caused more than 80% cell death. The concentration at which CA is 50% cytotoxic (CC50) value was 44.19. Since a concentration of 7.8 µM showed toxicity to less than 20% of the cells, a maximum concentration of 8 µM was used for antiviral experiments (Fig. 1a).

### Effect of CA on the infection and replication of DENV

**Figure 2:**
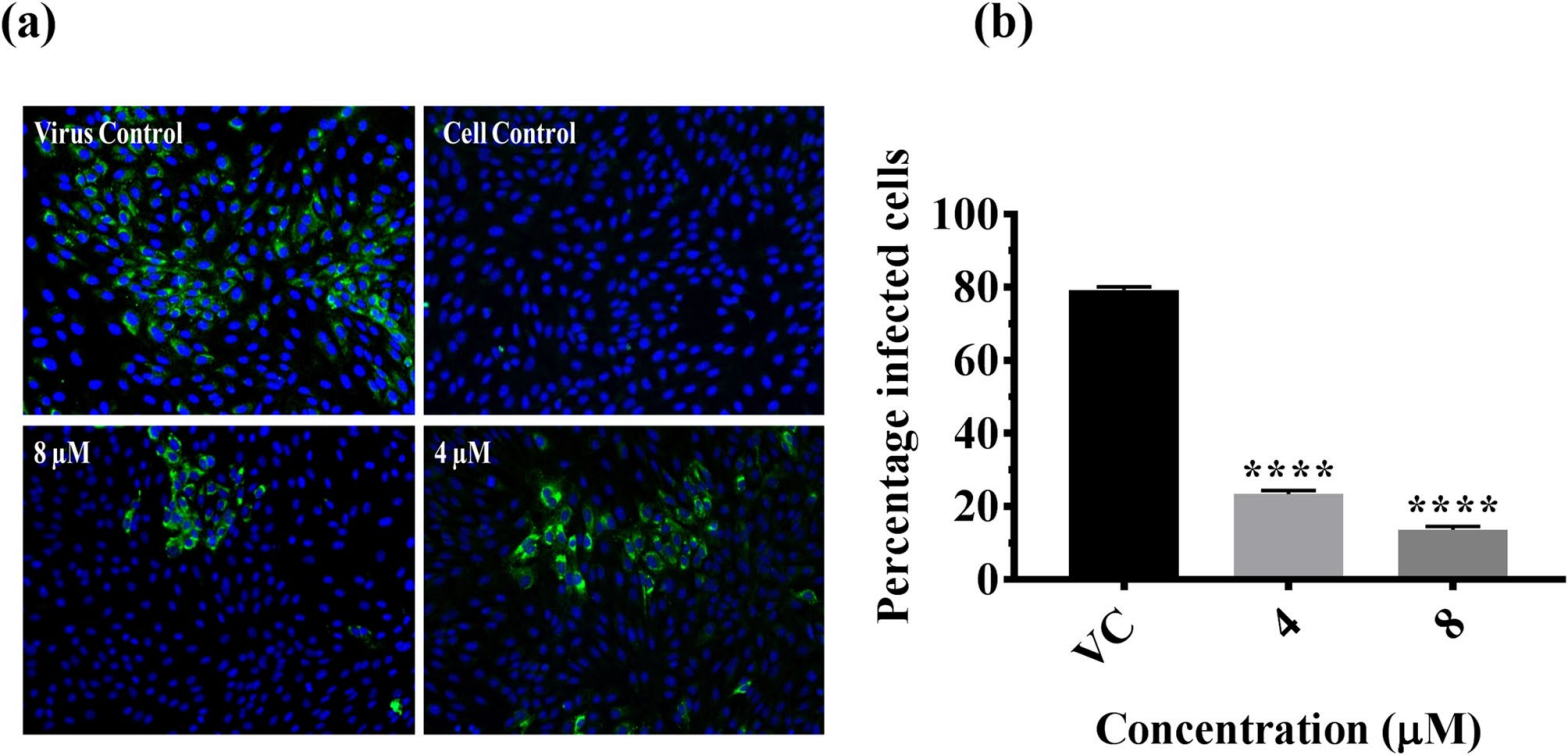
Direct virucidal effect of chebulinic acid (CA) on DENV-2 infection in Vero E6 cells. (**a**) Immunofluorescence staining images of DENV-2 infected Vero E6 cell lines at 72 h post-infection under co-treatment conditions. The cell nucleus was stained with DAPI (blue), and the DENV proteins in the infected cells were stained with FITC (green). (**b**) The quantitative depiction of the infected cell fluorescence levels from the microscopy image by Image J represented as percentage of infected-cells in comparison to the virus control. All the values are expressed as mean ± SEM of three experiments ****P < 0.0001 vs. control.

**Figure 3:**
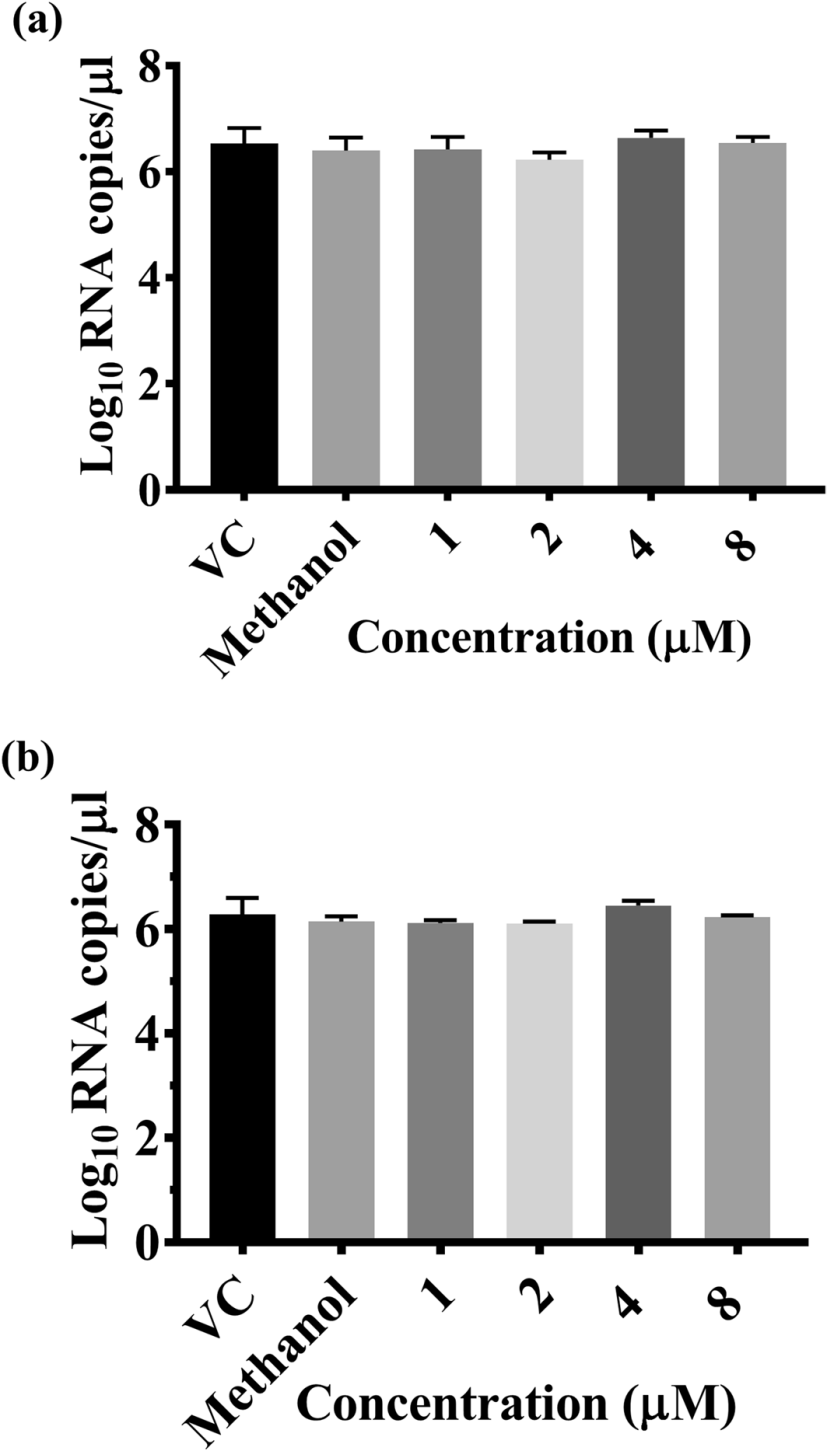
(**a**) Effect of pre-treatment of Vero E6 cells with CA on dengue virus infection and replication expressed in terms of log_10_ viral RNA copy numbers. (**b**) Effect of treatment of Vero E6 cells with CA post-infection on dengue virus infection and replication is expressed in log_10_ viral RNA copy numbers. VC refers to virus control in which no CA was added.

The effect of CA on DENV-2 was assessed using three methods: (i) Co-treatment of the virus with the CA followed by infection; (ii) pre-treatment of the cells before infection; and (iii) treatment of the cells post-infection. When the virus was co-treated with the compound for 1 h and used for infection, a significant reduction in the mean log_10_ titer of viral RNA/µL was observed at 120 h post infection with 8 µM concentration as compared to virus control (P < 0.05) (Fig. 1b). A dose-dependent reduction in the titer of the infectious virus particles, represented as log_10_ focus forming units (FFU/mL), was observed, which was found to be more prominent at both 4 and 8 µM concentrations (P < 0.0001) (Fig. 1c). Compared to the virus control (5.95 log_10_FFU/mL), a two log reduction was observed at 8 µM concentration (4.0 log_10_FFU/ml) (P < 0.0001), whereas a decrease of about 1.5 log_10_ was observed at 4 µM concentration (4.11 log_10_FFU/mL) (P < 0.0001). The percentage inhibition of the production of infectious virus particles was ~ 98% compared to the virus control at 8 µM concentration, and the half-maximum-inhibitory-concentration (IC50) value based on FFU data was 0.29 µM (Fig. 1d). Methanol, used to dissolve the CA had a mild inhibitory effect on infectious virus particle production (5.3 log_10_FFU/ml) at 8 µM but not on viral RNA titer (Fig. 1b,c). However, compared to methanol, 8 µM and 4 µM CA reduced virus titre by 93% and 92% respectively (P < 0.0001 for both concentrations vs methanol).To further confirm the antiviral activity of CA under co-treatment conditions, the percentage of infection following treatment was also assessed using an immunofluorescent assay at 72 h post infection. The average percentage of infected-cells in virus control were 79.25%, whereas, in the presence of CA at 4 μM and 8 μM concentrations, the percentage dropped to 23.3% (P < 0.0001)and 13.63% (P < 0.0001), respectively (Fig. 2). These results indicated that inhibition of the infection by CA occurred at the virus entry stage. The CA was found to have no effect on the replication of the virus as assessed by log_10_ viral RNA copies/µL when used for pre or post infection treatment (P > 0.05) (Fig. 3a,b).

### Effect of CA on the infection and replication of CHIKV

**Figure 4:**
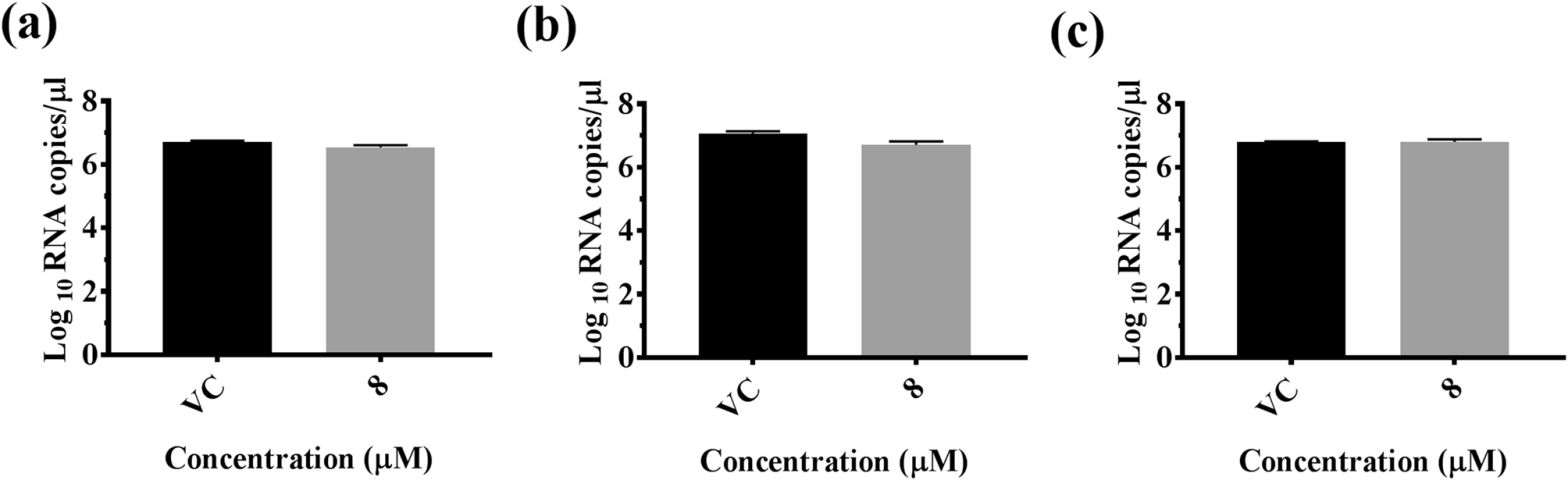
The effect of CA on CHIKV RNA levels in Vero cells, quantified by qRT-PCR under (**a**) pre-treatment, (**b**) co-treatment of virus with CA during infection, and (**c**) post-treatment conditions. The viral RNA titer was expressed as mean Log_10_ viral RNA copies/µL ± SEM of three experiments.

To screen for the antiviral activity of CA against CHIKV, a maximum non-toxic dose (8 µM) was used. The CA, even at a concentration of 8 µM, did not affect CHIKV infection or replication, as assessed by log_10_ viral RNA copies/µL under conditions of co-treatment of the virus with the CA before infection, pre-treatment of cells before infection, or treatment of cells post-infection (Fig. 4a–c).

### Docking studies involving E glycoprotein of DENV and CA

**Figure 5:**
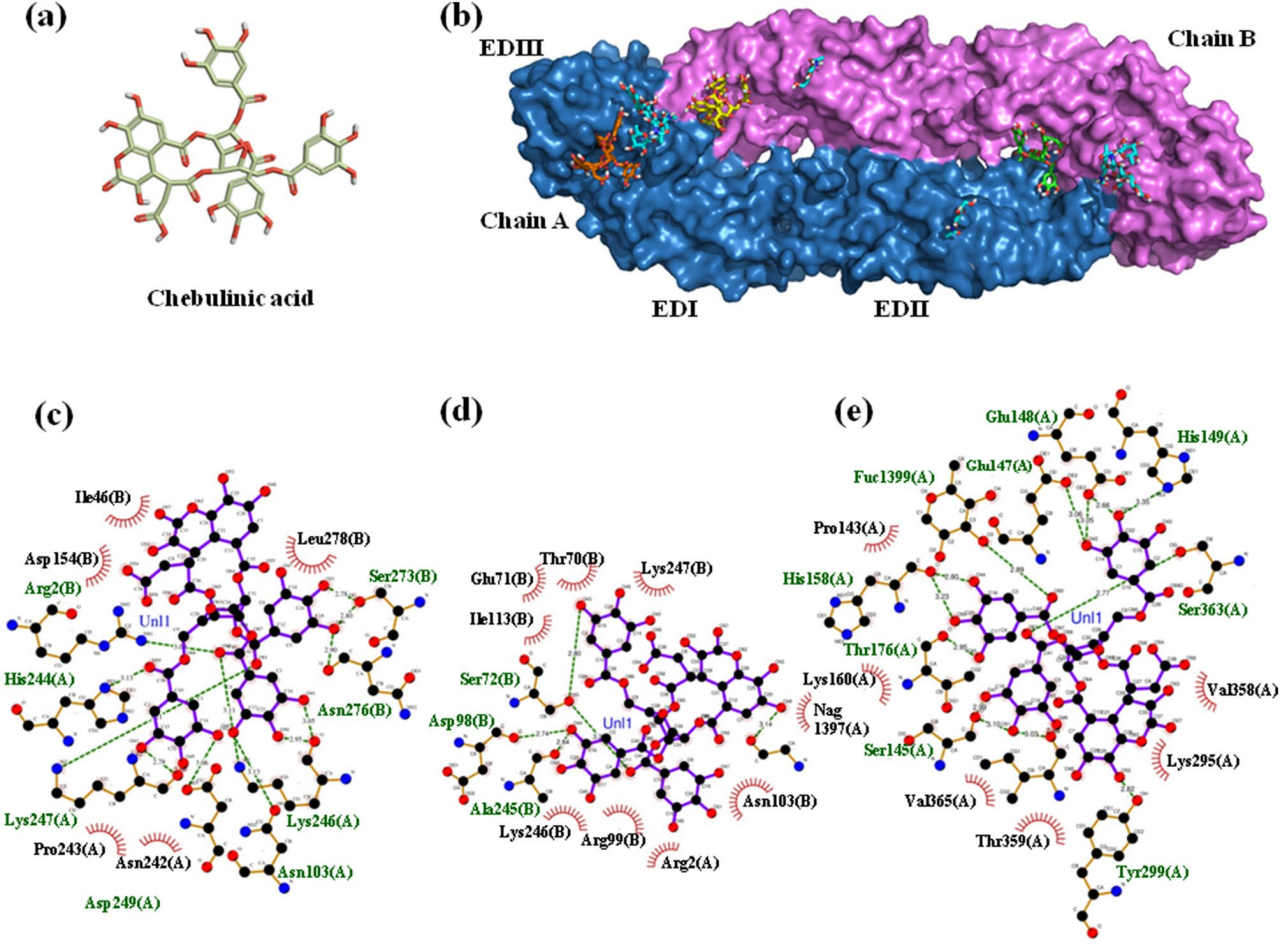
Structural depiction of chebulinic acid, DENV E protein homodimer, and their docked conformations. (**a**) Two-dimensional structure of chebulinic acid (CA), wherein the carbon, oxygen, and hydrogen atoms are depicted in olive green, red, and white colors, respectively. (**b**) Surface diagram of DENV E homodimer with docked conformations of CA molecules. The backbone of the homodimer chains A and B are represented in blue and pink colors, respectively. The glycans on the surface of E protein are represented in sticks with carbon backbone depicted in cyan color. The CA molecules docked at Kl β-hairpin loop, fusion peptide, and EDIII-EDI pockets are shown in sticks with green, yellow, and orange carbon color schemes. The oxygen and hydrogen atoms of the CA are depicted in red and white colors, respectively. This diagram has been prepared using the molecular visualization system PyMOL^47^. Schematic depiction of DENV E-CA interactions at (**c**) Kl β-hairpin loop pocket, (**d**) fusion peptide pocket, (**e**) EDIII-EDI pocket that is mediated by hydrogen bonds (dashed green lines) and by hydrophobic contacts (red arcs with spokes). These diagrams are generated by the LigPlot software^45,46^.

In its homodimeric form, the E protein of DENV is exposed on the viral surface and serves as a ligand to various host receptors. In this study, the blind and focused docking of E protein with CA was performed using the Autodock Vina^37^ software (Fig. 5a), which has been widely used for similar docking studies with DENV E protein^38–40^. Blind docking was performed with E protein homodimer and CA, followed by focused docking at multiple sites; (i) EDIII and the flexible stretch of amino acid residues joining EDI and EDIII involved in binding the GAGs receptor^41,42^; (ii) fusion loop residues (V382, E383, P384, G385) and lysine residues (K291, K295, K305, K307, K310) present in the flexible hinge between EDIII and EDI; (iii) fusion peptide (residues 100–108 on EDII)^11^; (iv) K1 β-hairpin loop (residues 268–280)^43^ and (v) glycosylation sites^44^. The physical locations of these sites on the E protein homodimer have been depicted in Supplementary Information (SI) Fig. S1. The docking results were further analyzed to identify the interacting residues and the types of interactions, using the LigPlot software^45,46^ (version 2.2) and visualized using the PyMOL software^47^ (version 2.3.3). CA showed binding to three significant regions of E protein, reportedly involved in binding to the primary receptors, GAGs, DC-SIGN, mannose receptor^48^, and facilitating the fusion process. Blind docking revealed a pocket at the interface of the two subunits of the homodimer, formed between the distal end of EDII of chain-A and EDI and EDII of chain-B (Fig. 5b). In this pocket, CA made contact with EDI and EDII of chain B, primarily with the residues of the K1 β-hairpin loop; whereas the rest of the contact residues are contributed by EDII of chain A (Fig. 5b,c). This CA-E protein interaction is mediated by 14 hydrogen bonds and 5 hydrophobic interactions (SI, Table S1). The second pocket involved in CA binding is primarily constituted of chain B and includes the residues of fusion peptide (Fig. 5b,d), wherein the binding is predominantly mediated by non-polar interactions with a total of 9 hydrophobic interactions and an additional 5 hydrogen bonds (SI, Table S1). CA also showed binding to these sites when tested individually during focused docking (data not shown).

The third region that CA showed binding to, encompasses EDIII; the associated hinge (the flexible region between DIII and DI); and the DI of chain A (Fig. 5b). CA showed binding to this region in blind as well as focused docking, wherein it bound the residue K295 (conserved across all four serotypes of DENV) and the sugar moiety (Fuc1399) attached to the residue N153 (Fig. 5e). This glycosylation site is also conserved in all the four strains of DENV. In this pocket, CA interacted with E protein through 14 hydrogen bonds and 6 hydrophobic interactions (SI, Table S1). On the other hand, CA did not show binding to the fusion loop, which is located deep inside domain III of E protein, in either of the docking experiments.

Interestingly, CA bound all the regions of E protein mentioned above with equivalent affinity, with the binding energies ranging from − 8.7 to − 8.2 kcal/mol. To further validate the docking results, the electrostatic complementarity (EC), another crucial indicator of specific interaction between ligand and a protein, was assessed using the FLARE software^49^. The surface maps generated using the software illustrate the positive, negative, and the no complementarity EC values for ligand and the protein E (SI, Fig. S2) and also depict the interacting residues (SI, Table S1). The interacting surfaces of the ligand and the protein showed considerable levels of electrostatic complementarity, supporting our docking results. In addition to the above mentioned receptors of DENV, prohibitin 1/2 has recently been shown to mediate DENV-3 entry into human neuroblastoma (SH-SY5Y) and microglia (CHME-3) cells^50^. Earlier, prohibitin-2 has been shown as a mediator of DENV-2 entry into insect cells^51^, whereas in another study the Vero cells have been shown to express prohibitin-1 on its surface^24^. However, it is not entirely clear if prohibitin-2 is also expressed on the surface of Vero cells. Nonetheless, given the status of prohibitins being ubiquitously expressed on many types of cells^52^, prohibitin-2 was presumed to be expressed on the surface of Vero cells and included in the in silico experiments that analyzed its role in DENV-2 host entry. In order to investigate if prohibitin-1/2 interact with DENV-2 E protein, protein–protein docking of modelled structures of prohibitin-1/2 and crystal structure of the coiled coil fragment of prohibitin-2 was set with E proteins of DENV-2/3. The structure of prohibitin-1 was modelled using server iTasser^53^ and SWISS-MODEL^54^ (SI, Fig. S3, Fig. 7a). The models thus generated were validated by comparison with other prohibitin modelled structures, retrieved from AlphaFold Protein Structure Database^55^ using the PyMOL software^47^. The SWISS-MODEL^54^ and iTasser^53^ structures of prohibitin-1 superimposed with other AlphaFold models (from distinct source organisms) with RMSD values of 4.4 and 16.6, respectively (SI, Fig. S3). Therefore prohibitin-1 and 2 structures, modelled using the SWISS-MODEL software^54^, were subjected to docking using the ClusPro software^56^. A total of five blind docking experiments were set up: (i) prohibitin-1 with DENV-2 E protein, (ii) prohibitin-1 with DENV-3 E protein, (iii) prohibitin-2 with DENV-2 E protein, (iv) coiled-coil fragment crystal structure of prohibitin-2 with DENV-2 E protein and (v) coiled-coil fragment crystal structure of prohibitin-2 with DENV-3 E protein, and the data was analyzed using the PyMOL software^47^. As shown in SI Fig. S4, none of the prohibitins showed binding on the top, exposed surface of the E protein homodimer, and instead showed binding on the surface that faces the lipid envelop of the virion. The face of the E homodimer that faces the environment was determined by superimposing the docked structures with another DENV-2 and CA docked structure, wherein the bound CA and other surface glycan moieties determined the exposed surface. These in silico studies remained inconclusive on the interaction between the E protein and the receptor prohibitin molecules. Further experimentation is required to establish the mode of interaction between the two species.

### Docking studies involving E glycoprotein heterodimer of CHIKV, host cell receptor, and CA

**Figure 6:**
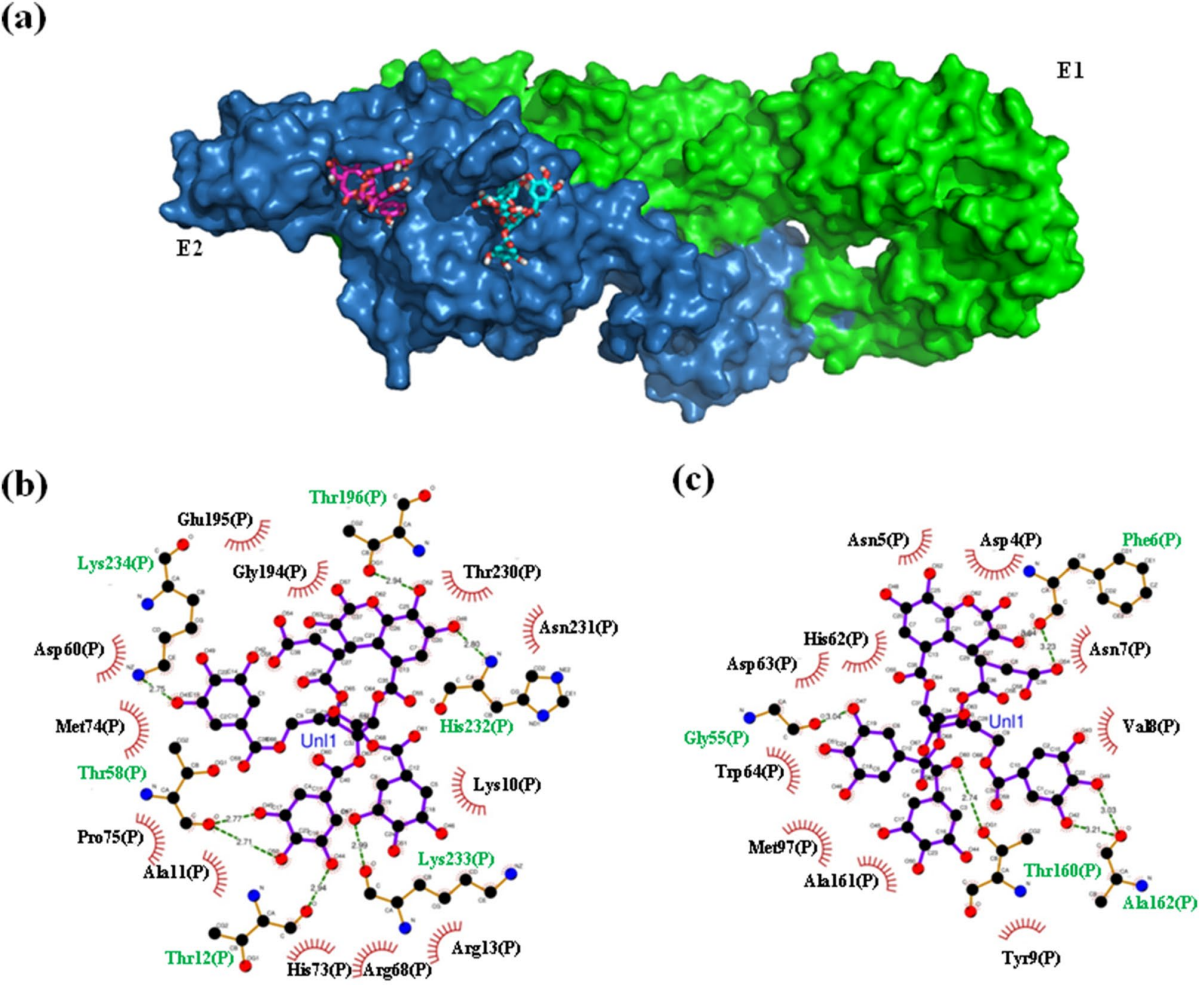
Structural depiction of CHIKV envelope glycoprotein E1-E2 heterodimer and chebulinic acid in their docked conformations. (**a**) Surface diagram of CHIKV E1-E2 heterodimer with docked conformations of CA molecules. The two chains of the heterodimer, E1 and E2, are represented in green and blue colors, respectively. The CA molecules bound at the pockets located at domain A and B interface of E2, and N-terminus of E2 within domain A, are depicted in sticks with carbon color scheme of pink and cyan, respectively. The oxygen and hydrogen atoms of the CA molecules are represented in red and white colors, respectively. This diagram has been prepared using the molecular visualization system PyMOL^47^. Schematic depiction of CHIKV E2-CA interactions at (**b**) pocket located at domain A and B interface of E2 (**c**) pocket located at the N-terminus of E2 within domain A, those are mediated by hydrogen bonds (dashed green lines) and by hydrophobic contacts (red arcs with spokes). These diagrams are generated by software LigPlot^45,46^.

**Figure 7:**
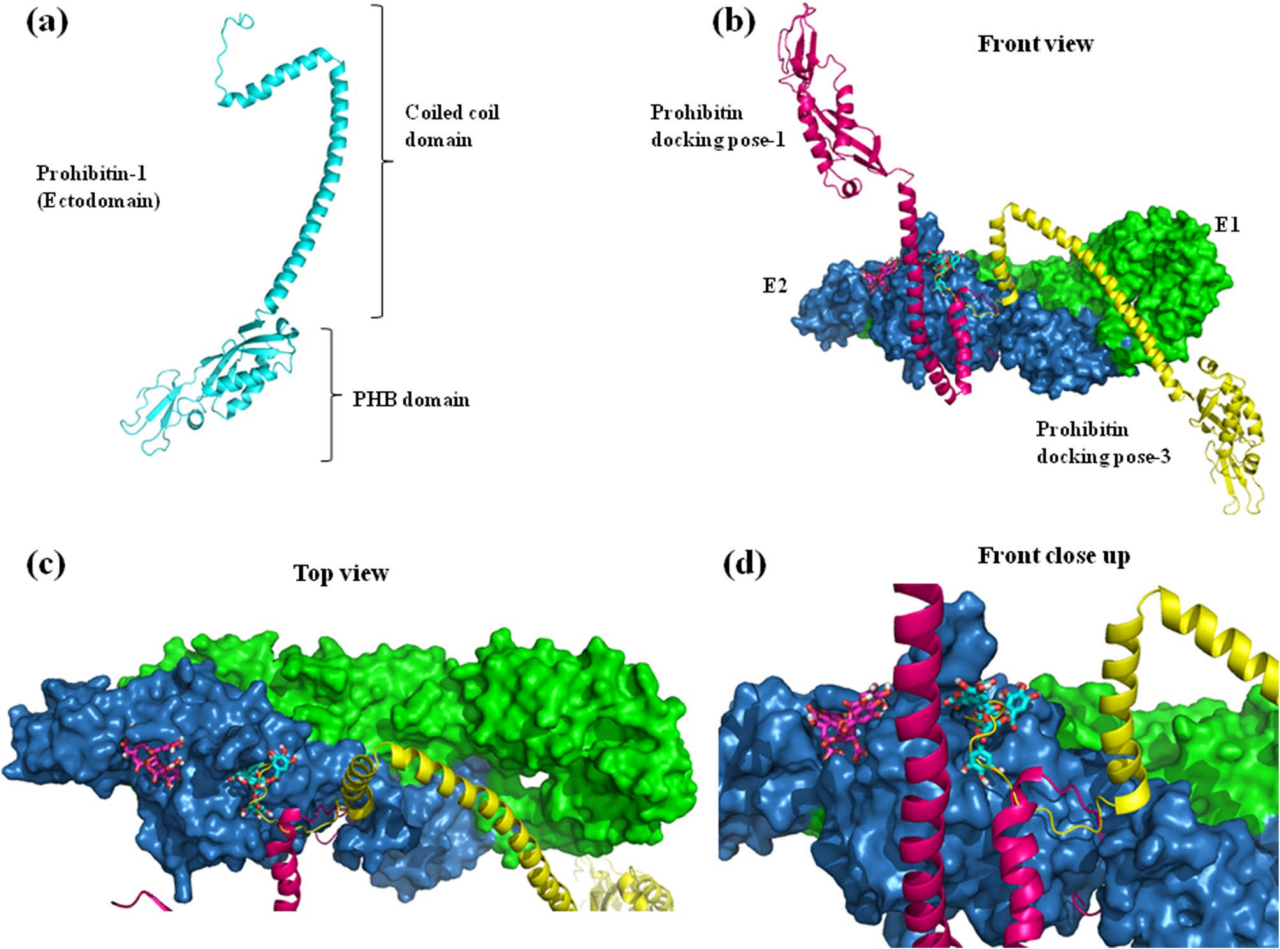
Structural depiction of prohibitin-1, CHIKV envelope glycoprotein E1-E2 heterodimer and their docked conformations. (**a**) Model structure of prohibitin ectodomain, generated using the SWISS-MODEL software^54^. The protein is depicted in cartoon presentation of protein backbone (cyan), showing the N-terminal prohibitin (PHB) and C-terminal coiled coil domains. (**b**) Front view of the docked conformations of prohibitin and CHIKV E1-E2 heterodimer. The E1 and E2 subunits are depicted in surface presentation with green and blue color schemes, respectively. The first and third docked conformations of prohibitin are shown in cartoon presentation in magenta and yellow color schemes. (**c**) The top view of the docked conformations of prohibitin and CHIKV E1-E2 heterodimer. (**d**) The close-up of the front view of the docked conformations of prohibitin and CHIKV E1–E2 heterodimer. These diagrams have been prepared using the molecular visualization system PyMOL^47^.

The crystal structure of the heterodimer of CHIKV E glycoproteins 1 and 2 (E1, E2) was also subjected to blind and focused docking using the same protocol as mentioned above. The entire structure of the E1/E2 heterodimer was used for the blind dock (SI, Fig. S5). CA was found occupying a junction pocket, formed within the E1 and E2 interface of the heterodimer, which has not been reported to play any significant role in virus attachment or fusion. Subsequently, focused docking was performed with the structure of E2 that was modelled using server iTasser^53^ because of the unavailability of the first three residues of E2 protein in the crystal structure (PDB ID 3N44) used for blind docking. The focused docking was performed using domains A and B of E2 (dAE2/dBE2) and CA, wherein the CA and E2 domains were observed interacting with binding energy in the range of − 8.1 to − 7.2 kcal/mol. CA bound E2 within domain A, through 6 hydrogen bonds and 10 hydrophobic interactions, whereas at another pocket that was formed at the interface of domains A and B, the interaction was mediated through 7 hydrogen bonds and 12 hydrophobic interactions (Fig. 6a). The residues of E2, involved in forming close contacts with the ligand, at these two distinct sites were analyzed using the LigPlot software^45,46^ (Fig. 6b,c). The details of the interacting residues and the type of interactions are listed in SI, Table S1. The research group of Sahoo and Chaudhary has recently reported the heparan sulfate (HS), binding motif (residues 104–109) in E2 protein and the critical residues, R104 and K107 (from within the motif) and residue R144 (from outside the motif), that are involved in binding the receptor. Comparison of these HS binding residues with CA binding residues of E2 (SI, Table S1), revealed no overlap between the two sites. In addition to HS, Vero cells have been shown to express another receptor of CHIKV, the prohibitin-1 protein. The site on E2 for prohibitin-1 binding has yet not been defined. Therefore, to identify the prohibitin-binding-site on E2 and correlate CA's activity through this site, the prohibitin-1 and E2 docking was performed. The structure of prohibitin-1 was modelled using SWISS-MODEL^54^ (SI, Fig. S3, Fig. 7a). The modelled structures of prohibitin-1 and E2 proteins were subjected to docking using the ClusPro software^56^, whereas the interface residues were analyzed using the PDBePISA software^56^. The AutoDock Vina/LigPlot and ClusPro/PISA data of E2-CA and E2-prohibitin-1 docking were analyzed using the PyMOL software^47^ to assess the CA and prohibitin-1 binding sites on E2 (Fig. 7b). In the first two conformations, prohibitin-1 interacted with E2 through a deep and narrow pocket, formed at the interface of domains A and C of E2 (Fig. 7b–d), and herein, the prohibitin-1 binding site did not overlap with the CA binding site of E2. However, in the third (exclusive) conformation, the coiled-coil domain of prohibitin-1 did not traverse deep into the pocket but bound it at one of the edges of the pocket, whereas it’s C-terminal end extended into the CA binding site, showing an overlap between the two sites (Fig. 7b–d). Therefore, the region of E2 that was bound by the first and third conformers of prohibitin-1, define its binding site, starting from the dAE2/dCE2 interface pocket and extending into the CA binding pocket in the dAE2. The residues involved in making protein–protein contacts at the interface of E2-prohibitin-1 complex are listed in SI, Table S1.

## Discussion

Dengue is an arthropod-borne viral disease. During the last 10–12 years, various small molecules and repurposed drugs like anti-malarial drug chloroquine^57^, anti-inflammatory drugs such as prednisolone^58^ and lovastatin^59^; iminosugar, celgosivir^60^, anti-parasitic drug ivermectin^61^; nucleoside analog, balapiravir^62^ and others had been tested against dengue; however, none could be developed as a potential drug candidate against DENV. Since, the small molecule/drug candidates did not perform as desired in clinical trials^63^, scientists turned towards natural products, phytochemicals, for finding therapeutic agents against dengue. More than 70 medicinal plants have been identified so far, which show anti DENV activity, effected through extracts and their purified compounds^25^.

In the present study, we report chebulinic acid (CA), a natural product isolated initially from *Terminalia chebula* flower extract^31^, as the natural anti-DENV phytochemical. Co-treatment of DENV-2 with CA before infection, reduced the production of infectious virus particles by a factor of 2 logs at 8 µM concentration and 1.5 logs at 4 µM concentration, and the percentage inhibition achieved was greater than 98% with an IC50 value of 0.29 µM. In the case of DENV RNA, the reduction was observed only at 8 µM concentration. Real-time RT-PCR quantifies viral RNA from both infectious and non-infectious virus particles, and the cells infected with DENV produce immature virus particles which are not infectious^64^. Moreover, one-step real-time RT-PCR amplifies both positive and negative strand viral RNA in the culture supernatant. Thus, real-time RT-PCR being a sensitive assay, might have decreased the fold difference in viral RNA load between the treatment and virus control thereby affecting the statistical significance. Methanol used to dissolve the CA had a mild inhibitory effect on infectious virus particle production but not on viral RNA titre. It is possible that methanol might have mild toxic effects on cells thereby affecting the virus production. However, CA had significant inhibitory effect on the virus titre compared to methanol, suggesting that the inhibitory effect is not due to methanol but CA. The in vitro data obtained in the current study shows significant correlation with the findings of an earlier study, where antiviral activity of CA was demonstrated against HSV-2^31^. Alike HSV-2 inhibition at the attachment and penetration stage, CA inhibited the DENV-2 infection when co-treated with DENV-2 prior to infection, whereas, had no effect on viral entry and resulting infection when used to treat cells before and after the infection. The IC50 values of CA for HSV-2 and DENV-2 were also found somewhat comparable at 0.06 ± 0.002 μg/mL (0.06 µM) and 0.29 µM, respectively, given the fact that both the viruses are unrelated and use different glycoproteins to make contact with the host.

On the other hand, CA was found ineffective against CHIKV infection suggesting its failure to block the entry step of CHIKV infection in Vero cells. The results obtained from the in vitro DENV-2 inhibition assays lead to the hypothesis that CA functions through an active host-entry-inhibition mechanism. To further investigate this hypothesis, the in silico docking of glycoproteins of DENV-2 and CHIKV was performed with CA using the Autodock Vina software^37^. In the case of DENV, CA showed binding to three crucial sites: (i) K1 β-hairpin loop; (ii) fusion peptide; and (iii) a pocket formed by the residues of EDIII, the flexible hinge connecting EDIII and EDI and EDI. In an earlier study, the conformational shifts in K1 β-hairpin channels have been demonstrated to initiate the fusion process of virus and host membranes^43^. CA binding of the K1 β-hairpin loop residues may interfere in this conformational change resulting in the inhibition of the fusion process, which is an essential step in the virus entry. In another study, the interface between the DENV E protein and its receptor DC-SIGN (involving 36 residues of E protein) was elucidated^65^. CA was found to bind this region of E-protein through 13 residues, out of which seven residues (T70, E71, G102, N103, A245, K246, K247) are common to E2-DC-SIGN interface^65^ and include residues G102 and N103 of the fusion peptide. Therefore, CA bound in the fusion peptide pocket of E protein may interfere with the DENV-receptor attachment and the fusion process. In the third pocket, involving EDIII and EDI, CA showed binding to a residue K295 (conserved across the four serotypes of DENV), which is reported to directly bind to GAG receptor^42^, suggesting that CA bound in this region may block the interaction between E protein and the receptor.

Furthermore, in the same cavity, CA shows binding to the sugar moiety (Fuc1399) attached to glycosylation site residue, N153, which is also conserved in all the four strains of DENV and is reported to play a role in binding mannose receptor on host cells^12,48^. CA bound in this region may inhibit DENV infection mediated through the mannose receptor. Thus, in vitro and in silico data correlates with one another and collectively demonstrates the potential of CA to bind on distinct sites on E protein, possibly inhibiting multiple types of virus-host interactions involved in receptor attachment and fusion process.

In the present study, the inhibitory activity of CA against DENV-2, assessed in Vero cells, was considered to be directed against receptor GAGs and co-receptor glycosphingolipid nLc4Cer^14,24^. The docking studies with the co-receptor nLc4Cer were not performed due to its small size (smaller than CA), which may have behaved as a ligand in the docking experiments. Being a polyol, the exposed polar head of the glycosphingolipid is chemically very similar to CA. Therefore, we hypothesized that both might be binding to similar sites on the E protein surface. In addition to the above mentioned receptors of DENV, prohibitin-1/2 have been recently shown to mediate DENV-3 entry into human neuroblastoma (SH-SY5Y) and microglia (CHME-3) cells^50^. Also, prohibitin-2 has been shown earlier as a mediator of DENV-2 entry but in the insect cells^51^. On the other hand, Vero cells have been shown to express prohibitin-1 on its surface^24^, however, the study doesn’t say anything about prohibitin-2 expression and there are no reports stating otherwise. Therefore, given the fact that prohibitins are ubiquitously expressed on various cells^52^ and the fact that DENV-3 showed binding to prohibitin-1/2, docking experiments with DENV-2 E protein and prohibitins were conceptualized using DENV-3 E protein as a positive control. DENV-2/3 E proteins docking with prohibitin-1/2 did not show any binding on the exposed surface of the E homodimers. Since the coiled coil domain of prohibitins have been shown to be involved in physiological binding and signalling^66^, docking of DENV-2/3 E proteins was performed with the crystal structure of coiled coil fragment of prohibitin-2. Herein also, the prohibitin coiled coil fragment did not show binding on the desire surface of the DENV E proteins. These results led us to hypothesize about what could be the mode of interaction of these proteins. Prohibitins have been shown to exist in monomeric, dimeric and oligomeric forms on the surface of cells and the organelles^67^, therefore, possibly prohibitin-1/2 do not bind E protein of DENV in their monomeric state, and recognize binding sites that may appear at the interface of the heterodimers or oligomers. Secondly, during fusion process of DENV with host cells, the E protein dimmers undergo significant conformational changes, wherein the domains of E protein bends over on itself, resulting in exposure of the otherwise obscured surface of E homodimers^68^, the surface for which the prohibitin-1/2 showed affinity in docking experiments. This lead us to hypothesize that may be prohibitins do not make the first contact with virus and probably by binding to the freshly exposed regions in the middle of a fusion process, they assist in fusion of the two membranes, thereby mediating a successful infection, although, proving these hypotheses would require further studies.

Another fascinating mechanism of DENV infection has been reported recently, wherein the DENV-2 E protein was shown to interact co-operatively with multiple receptors expressed on a single cell for a successful invasion. Lo, et al. have shown a cooperative interaction between mannose receptor, DC-SIGN, and CLEC5A (C-Type Lectin Domain Containing 5A) signaling receptor, where the virus is first recruited through interaction with the former two receptors, which later involve CLEC5A to form a hetero-complex facilitating entry as well as activation of the host cell to produce cytokines^69^. These observations further signify the importance of multi-receptor targeting to inhibit the DENV infection effectively, as has been hypothesized in the case of CA activity. Therefore, to fully realize CA's true potential as an efficacious anti-DENV drug molecule, further investigations into its inhibitory activity against other serotypes, employing different cell types, exhausting the entire range of DENV receptors are required. Therefore, by binding to sites of different types of receptors of DENV, expressed differentially on different cells, CA may inhibit the establishment of infection through various cell types. Furthermore, binding of the CA with those previously mentioned three binding sites (having residues conserved in all four serotypes of DENV) indicates its potential as a pan DENV inhibitor.

In the case of CHIKV, receptors such as GAGs (heparin sulfate) and prohibitin expressed on Vero cells^24^ are believed to mediate the virus entry. The inability of CA to inhibit CHIKV infection was therefore attributed to its failure to bind sites of E2 that are involved in making contact with these host receptors. The HS binding motif in E2 protein, was deciphered recently by the research group of Sahoo and Chaudhary, defining its critical residues, R104 and K107 (from within the motif) and R144 (from outside the motif)^56^. Comparing HS binding residues with CA binding residues (SI, Table S1) on E2 showed them occupying mutually exclusive sites. This provided a possible explanation about the incapability of CA to inhibit CHIKV infection in Vero cells, which express HS (GAGs) on its surface. In addition to HS, Vero cells express another receptor prohibitin used by CHIKV for infection. However, the binding site of prohibitin on E2 was still elusive. Our study has identified the potential E2 binding site through E2-prohibitin docking studies. This analysis predicted five conformers of prohibitin-1, which interact with E2 through a deep and narrow pocket formed at the junction of E2 domains A and C. Out of these five conformations, only one (third best) conformation shows simultaneous binding to this pocket and the CA binding site (present in domain A of E2), whereas, the rest of the four conformations bound the pocket exclusively. This analysis suggested a possible explanation of the ineffectiveness of CA against CHIKV infection of Vero cell, as CA was observed blocking only one of the five contact sites of the prohibitin, allowing the virus to establish contact with the host receptor in its presence and cause infection. Kam et al. demonstrated the activity and mechanism of inhibition of a neutralizing antibody which recognized a linear epitope (E2EP3) on the E2 N-terminus, involving residues 3–10 as the core binding region^70^. Selvarajah et al. described the binding and inhibitory activity of C9 neutralizing monoclonal antibodies (nAb), which bound E2 protein in its acid-sensitive region through a critical residue A162, which plays a crucial role during virus fusion and entry into host cells^71^. These reported residues, 3–10 and residue A162, bound by these nAb on E2 surface, have been demonstrated in the current study to overlap with the E2-CA interface residues, indicating a possible inhibitory role of CA against a receptor other than HS and prohibitin-1. This further signifies the importance of simultaneous inhibition at multiple receptor-recognition-sites on the viral surface to effectively block these interactions and thus the infection. Therefore in vitro assays and the in silico studies with CA and glycoproteins of DENV2 and CHIKV helped decipher the possible mechanism employed by CA to inhibit infection of DENV2 in Vero cells, by directly blocking the virus-host interactions at receptor binding and fusion steps during infection.

The findings of the current study suggest that CA may inhibit DENV-2 entry into the different types of host cells and therefore may act as prophylactic/therapeutic agent against DENV-2. CA may act against the freely circulating viruses however, it might not be effective against the intracellular ones. Therefore, CA may be developed into a combination therapy, where, in conjunction with other direct inhibitors of the viral replication, it may allow successful disease management.

## Methods

### Maintenance of cell cultures

Vero E6 cells were grown in Modified Eagle's Medium (MEM, HiMedia) supplemented with 10% heat-inactivated fetal bovine serum (FBS). The cultures were maintained at 37 °C in a 5% CO_2_ humidified incubator. CHIKV strain (ECSA genotype) (strain number 61573, African genotype) and DENV-2 (strain number 803347) were used for the viral challenge.

### Preparation of virus stocks

The stocks of DENV-2 and CHIKV were produced by infecting C6/36 or Vero E6 cells and incubating at 37 °C with 5% CO_2_. After 3–5 days, depending on the development of cytopathic effects, the culture supernatants were harvested, and viral titers were determined using a focus forming unit (FFU) assay.

### Preparation of chebulinic acid stock solution

Chebulinic acid was dissolved in methanol to prepare a 10 mM stock from which required volume is further diluted in MEM to obtain the required concentration. The final concentration of methanol in 8 µM CA was less than 0.1%.

### Cell viability assay

The cytotoxicity of the compound, CA (Glentham life sciences, GK3169), was evaluated using the 3-(4,5-dimethythiazol-2-yl)-2,5-diphenyl tetrazolium bromide (MTT) reduction assay and microscopic examination in the Vero E6 cell lines. Cells were seeded in 96-well plates at an initial density of 2 × 10^4^ cells per well with concentrations of the compound from 500 to 1 µM and incubated for 120 h. After incubation, MTT solution (5 mg/mL) was added to the cells and kept for 3 h. The medium was then discarded, and 100 µL of acidified isopropanol (isopropanol in 5% 0.1 N HCl) was added to each well and incubated at 37 °C for 1 h. The readings were taken in a microplate reader (infinite F50, Tecan, Switzerland) at a wavelength of 570 nm with a reference filter at 690 nm. All the experiments were performed in triplicates in two to three independent trials. The proportion of dead cells or viable cells was calculated compared to cells treated with vehicle control.

### Antiviral activity

All the experiments were carried out in 24 well plates. For DENV-2, 5 × 10^4^ Vero cells per well were seeded in the plate and allowed to form a confluent monolayer for 24 h and infected with 0.1 multiplicity of infection (MOI) of the virus in 100 µL. For CHIKV, 2 × 10^5^ cells per well were seeded and allowed to form a confluent monolayer for 24 h and infected with 0.01 MOI of the virus in 100 µL. MOI was calculated based on the number of cells used for seeding. For seeding the plate, MEM with 10% FBS was used. After allowing adsorption for 1 h, the inoculum was removed and the cells were washed twice with PBS and MEM with 2% FBS (maintenance media) was added and incubated. To study the effect of pre-treatment on viral replication, the cells were pre-treated with the CA at 8 µM concentration for 24 h and then were infected. To investigate the effect of the CA post-infection, the cells were first infected and, after 1 h, were treated with 8 µM concentration of the compound. To study the direct effect of the CA on the viruses, DENV-2 or CHIKV were treated with 8 µM, 4 µM, 2 µM, and 1 µM concentrations of the compound for 1 h and were used to infect the Vero E6 cell line. After infection, cells were incubated for 24 h for CHIKV and 120 h for DENV, and culture supernatants were assessed by FFU assay for the number of infectious virus particles and real-time RT-PCR based assays for RNA copy number. All the experiments were performed in triplicates. Virus titers were expressed in log_10_ titers and compared between different treatment groups using one-way ANOVA. The P values were corrected for multiple comparisons.

### Focus forming unit (FFU) assay

The infectious virus titer in the culture supernatant was assessed as described earlier^38, 72^. Briefly, 2 × 10^4^ (for DENV) and 3.5 × 10^4^ (for CHIKV), Vero E6 cells in MEM with 10% FBS were seeded in a 96 well plate. After the cells form a confluent monolayer, the tenfold dilutions of DENV-2 or CHIKV virus from the culture supernatants were made, and 100 µL of different dilutions were used to infect the cells for one hr. After incubation, the inoculum was removed. Then 1.8% carboxy methyl cellulose diluted in 2 × MEM in 1: 1 ratio with a final concentration of 2% FBS was added, and the plate was incubated at 37 °C for 120 h for DENV-2 or 24 h for CHIKV. After incubation, cells were washed with PBS and fixed with chilled acetone and methanol in a 1:1 ratio for 15–20 min. After fixation, virus foci were developed using anti-DENV-2 E Antibody (1:250) or anti CHIKV antibody (1:300) followed by the addition of Goat anti-mouse IgG HRP conjugate (1:1000) and True Blue Peroxidase Substrate (KPL). Plates were incubated in the dark at RT for 20 min. Then the plates were dried and observed under a light microscope. The plates were scanned using a scanner at 600 dpi resolution (HP Scan jet G2410) for better observation. Blue color foci were counted in each well to calculate the virus titer and expressed as log_10_ FFU/mL.

### Quantitative RT-PCR

The effect of the CA on the production of viral RNA was assessed by quantitative RT-PCR. RNA from the cells and supernatants were extracted using QIAmp viral RNA minikit (QIAGEN, Valencia, CA) method following the manufacturer's instructions. Viral load was assessed in supernatant collected at 24 h for CHIKV and 120 h post-infection for DENV-2. The RT-PCR cycling conditions, the primers, and probes used to amplify and detect CHIKV and DENV-2 have been described earlier^38,72^. Tenfold dilutions of known concentration of in-vitro transcribed CHIKV and DENV-2 RNA were used as standards, and viral RNA copy was calculated. Log_10_ viral RNA copies number/µL was compared between cultures with different CA concentrations, and virus control and inhibitory activity were determined.

### Immunofluorescence assay

The quantitative estimation of the virus infectivity was performed using immunofluorescence assay (IFA) as described earlier with a minor modification^73^. To measure the infectivity, only co-treatment of cells was performed by incubating cells with different concentrations of CA with DENV-2. Approximately 5 × 10^4^ Vero E6 cells were seeded per well in a 24 well plate with a coverslip placed at the bottom. The cells were allowed to form the confluent layer while incubated in a 5% CO_2_ incubator at 37 °C. The coverslip was recovered three days post-infection, and cells were fixed by using chilled acetone and methanol in a 1:1 ratio and kept in the 4 °C for 15–20 min. Coverslips were then washed with PBST thrice and blocked with 1% BSA (Sigma-Aldrich St. Louis, MO, USA) in PBS and then incubated for 1 h at 37 °C. After incubation, primary antibody (1:20) (An anti dengue virus envelope antibody, E3F6) was added and followed by secondary antibody addition (1:1000) (Anti Mouse IgG FITC conjugated) (Sigma-Aldrich St. Louis, MO, USA) was added and incubated for 1 h. Afterward, the coverslips were mounted onto the slides with a drop of mowiol (mounting solution) containing 4′,6-diamidino-2-phenylindole, dihydrochloride (DAPI) (0.01 mg/mL) (Sigma-Aldrich St. Louis, MO, USA) (Nuclear stain). The slides were observed under a fluorescent microscope (EVOS Floid cell imaging station, Thermo Fisher Scientific, Bedford, MA, USA) with 20 × fixed lens magnification. The images were acquired using a combination of blue and green light filters. Approximately 5–6 fields per coverslip were observed, and cells were quantified by calculating the total number of cells and infected cells per field by splitting images with software ImageJ^74^ (version 1.51; https://imagej.nih.gov/ij/notes.html). Percentage infection thus calculated of each acquired image of Immunofluorescence assay.

### Structural modelling

Prohibitin is one of the receptors that mediate cellular binding and entry of CHIKV and DENV. To find out the binding site of E of DENV and residues of E2 of CHIKV interacting with the receptor, in silico docking studies with DENV E, CHIKV E2 and prohibitin were conceptualized. However, as the crystal structure of prohibitin-1/2 was not available, structure modeling was performed to attain the tertiary structure of the protein. Modeling was performed with the help of online modelling servers iTasser^53^ (https://zhanggroup.org/I-TASSER/) and SWISS-MODEL^54^ (https://swissmodel.expasy.org/interactive). For performing docking of E2 of CHIKV with CA and receptor prohibitin-1, the structure of E2 was also modelled using server iTasser^53^ as the crystal structure of E2 (PDB ID 3N44) does not feature the first three residues at its N-terminus. Three model structures of prohibitin-1 (source organisms: *Saccharomyces cerevisiae*, *Dictyostelium discoideum* and *Schizosaccharomyces pombe*), used for comparison with human prohibitin-1 models, were retrieved from AlphaFold Protein Structure Database^55^ (https://alphafold.ebi.ac.uk/).

### Receptor and ligand preparation

The three-dimensional structures of CHIKV surface glycoprotein E1/E2, DENV-2 and DENV-3 surface glycoprotein E, and coiled coil of prohibitin-2 were retrieved from the Protein Data Bank (PDB ID: 3N44, 1OAN, 1UZG and 6IQE, respectively). The structures of proteins like prohibitin-1/2 and E2 (CHIKV) were modeled online using modeling server iTasser^53^ and SWISS-MODEL^54^. Chosen structures were used as the receptor for molecular docking. The two-dimensional structure of CA was downloaded from the PubChem database (CID 72284) and converted into three-dimensional conformers by the docking software. The protein structure was kept rigid; meanwhile, the ligand was fully flexible. Subsequently, the protein was prepared for docking by removing the water molecules and adding the hydrogen atoms, followed by the minimization process. Root Mean Square Deviation (RMSD) of the protein crystal structures was also performed by superimposing the structures in the molecular graphics system, PyMOL^47^ (version 2.3.3; url:https://pymol.org/installers/PyMOL-2.3.3_0-Windows-x86_64.exe).

### Molecular docking and binding energy estimation

Molecular docking of 3D glycoprotein structures with CA was performed using Autodock Tools and Autodock Vina^37^ (version 1.1.2; https://mybiosoftware.com/autodock-vina-1-1-2-molecular-docking-virtual-screening-program.html). Auto Dock Tools were used to prepare protein and ligand structures by adding polar hydrogen bonds, optimizing the interactions between protein and ligand. A three-dimensional grid for DENV and CHIKV surface glycoprotein was designed with a size of 44 Å × 58 Å × 126 Å and 40 Å × 74 Å × 54 Å, respectively, along the X, Y, Z axis to define the search space for CA to be docked against glycoproteins. Blind docking was performed using the entire protein structure as the binding site for the ligand chebulinic acid. These docking results include the binding energy value given in Kcal/mol, hydrogen bond location and distance, and closely interacting residues. The docked PDB files were further analyzed using another software LigPlot^45,46^ (version 2.2; https://www.ebi.ac.uk/thornton-srv/software/LigPlus/download.html), which automatically generates schematic diagrams of protein–ligand interactions mediated by hydrogen bonds and hydrophobic contacts. The resulting diagrams depict hydrogen bonds by dashed lines between the interacting atoms. An arc illustrates the hydrophobic interactions with radiating spokes facing the interacting atoms, and the interacting atoms are represented with spokes facing the interacting residue. The electrostatic complementarity (EC) also plays an essential role in active site recognition and contributes significantly to free binding energy. For this analysis, the protein–ligand docked structures were first prepared using default settings of the software in the structure-based modelling suite FLARE^49^ (version 5.0.0; http://www.cresset-group.com/flare/). The limit of the size of the active site was chosen to be 3 angstroms to represent biological interactions. The surface maps generated from this suite illustrate the positive, negative, and no complementarity EC values in green, red, and white, respectively. All images were rendered in the modelling suite FLARE^49^.

To decipher the protein–protein interface, docking of the proteins, prohibitin-1/2, E of DENV-2, DENV-3 and CHIKV E2 was performed using the ClusPro software^56^ (version 2.0; https://clusphro.bu.edu/login.php). For this docking, modelled structures of prohibitin and CHIKV E2 were used. The docked PDB files generated by the ClusPro^56^ suite were analyzed using another software, PDBePISA^75^ (https://www.ebi.ac.uk/msd-srv/prot_int/cgi-bin/piserver). This interactive tool allows analysis of macromolecular interfaces, as it provides information about the interacting residues and the type of interactions between the two docked protein structures.

### Statistical analysis

In all the experiments, One way ANOVA followed by multiple comparisons between selected groups was used to find out the statistical significance. All the treatments were compared with virus control (VC). CC50 and IC50 values were calculated using regression analysis. A P value of less than 0.05 was considered significant. Statistical analysis was performed using GraphPad Prism software version 8.0.

## Supplementary Information

Supplementary Information.

## Data Availability

All data generated or analysed during this study are included in this published article [and its supplementary information files].
