# Supplementary material for: Studies on the antiviral activity of chebulinic acid against dengue and chikungunya viruses and in silico investigation of its mechanism of inhibition
Source: Sci Rep. 2022 Jun 21;12:10397. doi: 10.1038/s41598-022-13923-6 (PMC9213501; doi:10.1038/s41598-022-13923-6)
Supplement: Supplementary file 1 — Supplementary Information. [file 41598_2022_13923_MOESM1_ESM.pdf]

Studies on the antiviral activity of chebulinic acid against dengue and chikungunya viruses and *in silico* investigation of its mechanism of inhibition

Naiju Thomas[1], Poonam Patil[2], Anjana Sharma[1], Sandeep Kumar[1], Vikas Kumar Singh[1], Kalichamy Alagarasu[2], Deepti Parashar[2], Suman Tapryal[1*]

Author affiliations

[1] Department of Biotechnology, School of Life Sciences, Central University of Rajasthan, NH-8, Bandarsindri, Ajmer, Rajasthan 305817, India

[2] Dengue and Chikungunya Group, ICMR-National Institute of Virology, 20-A Dr. Ambedkar Road, Pune, Maharashtra 411001, India.

* Corresponding author

E-mail: suman_tapryal@curaj.ac.in

Supplementary Information

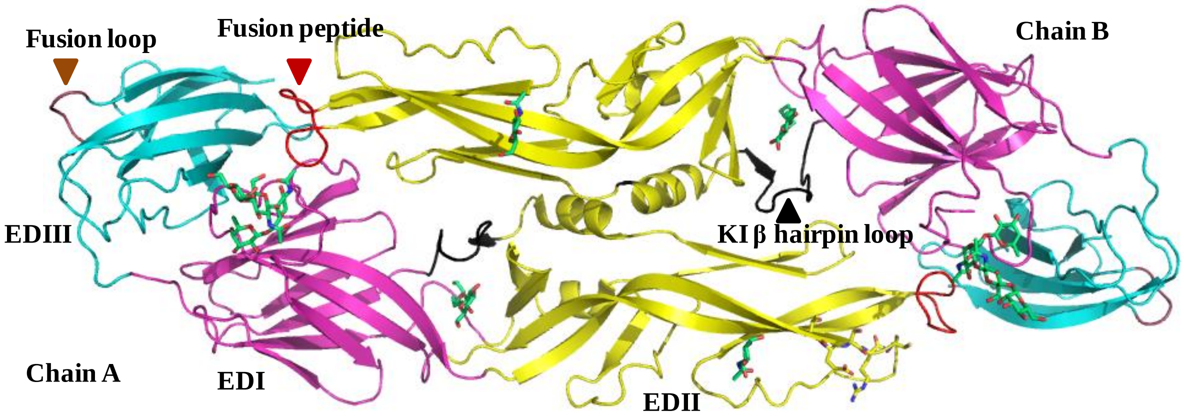

Figure S1: The crystal structure of the DENV-2 E protein homodimer (PDB ID: 1OAN) as it appears from the above in mature virions. The structural domains of E2 are represented in pink (EDI), yellow (EDII), and cyan (EDIII) colours. Important sites like the highly conserved fusion peptide, located at the distal end of EDII, is coloured in red; the glycans in EDI and EDII are represented as sticks (green); the Kl β-hairpin and fusion loops are represented in black and brown colours, respectively. The E protein structure analysis and image generation was performed using the molecular visualization system PyMOL[47].

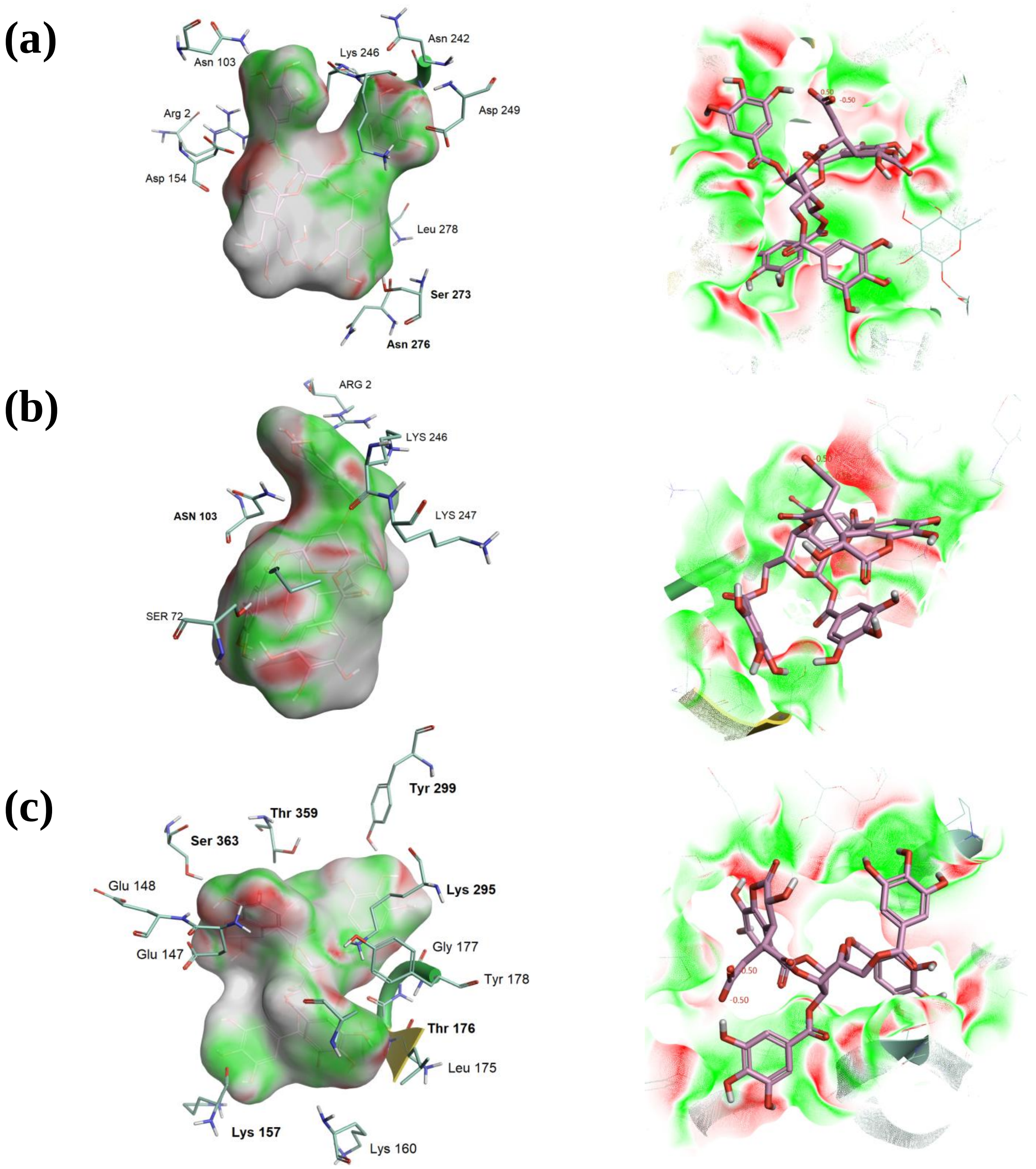

Figure S2: Electrostatic complementarity (EC) of Chebulinic acid and DENV E protein (PDB ID: 1OAN) complex. The electrostatic interactions between DENV E and CA were analyzed using the FLARE software[49]. The protein-ligand EC and surface complementarity (green = complementary, red = electrostatic clash, white= no complementarity) are shown on the ligand (left panel) and protein (right panel). The ligand and protein surface images were generated using the FLARE software[49].

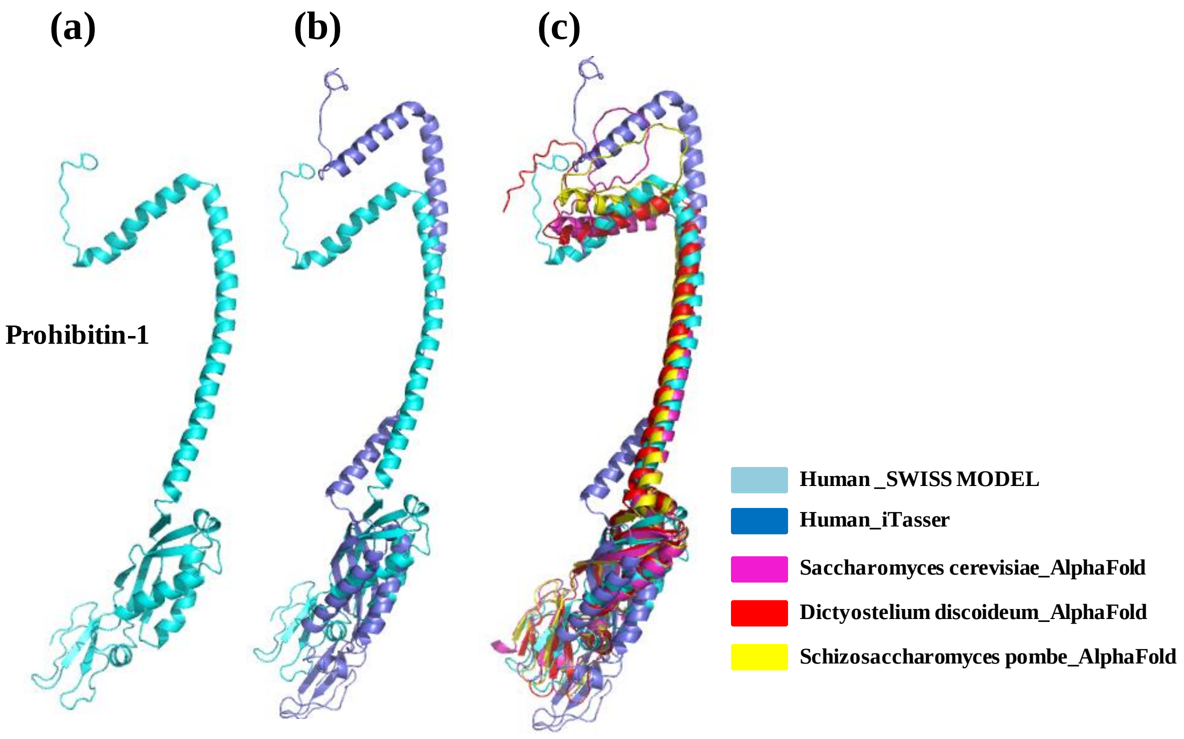

Figure S3: Superimposition and analysis of prohibitin-1, ectodomain structures modelled using suites such as SWISS-MODEL[54], iTasser[53] and that retrieved from database AlphaFold[55]. (a) Cartoon representation of human prohibitin-1. (b) Superimposed human prohibitin-1 proteins, modelled using two different software. (c) Superimposition of human, *Saccharomyces cerevisiae*, *Dictyostelium discoideum* and *Schizosaccharomyces pombe* prohibitin protein models. The structure analysis and the image genetation of prohibitin-1 models was performed using the molecular visualization system PyMOL[47].

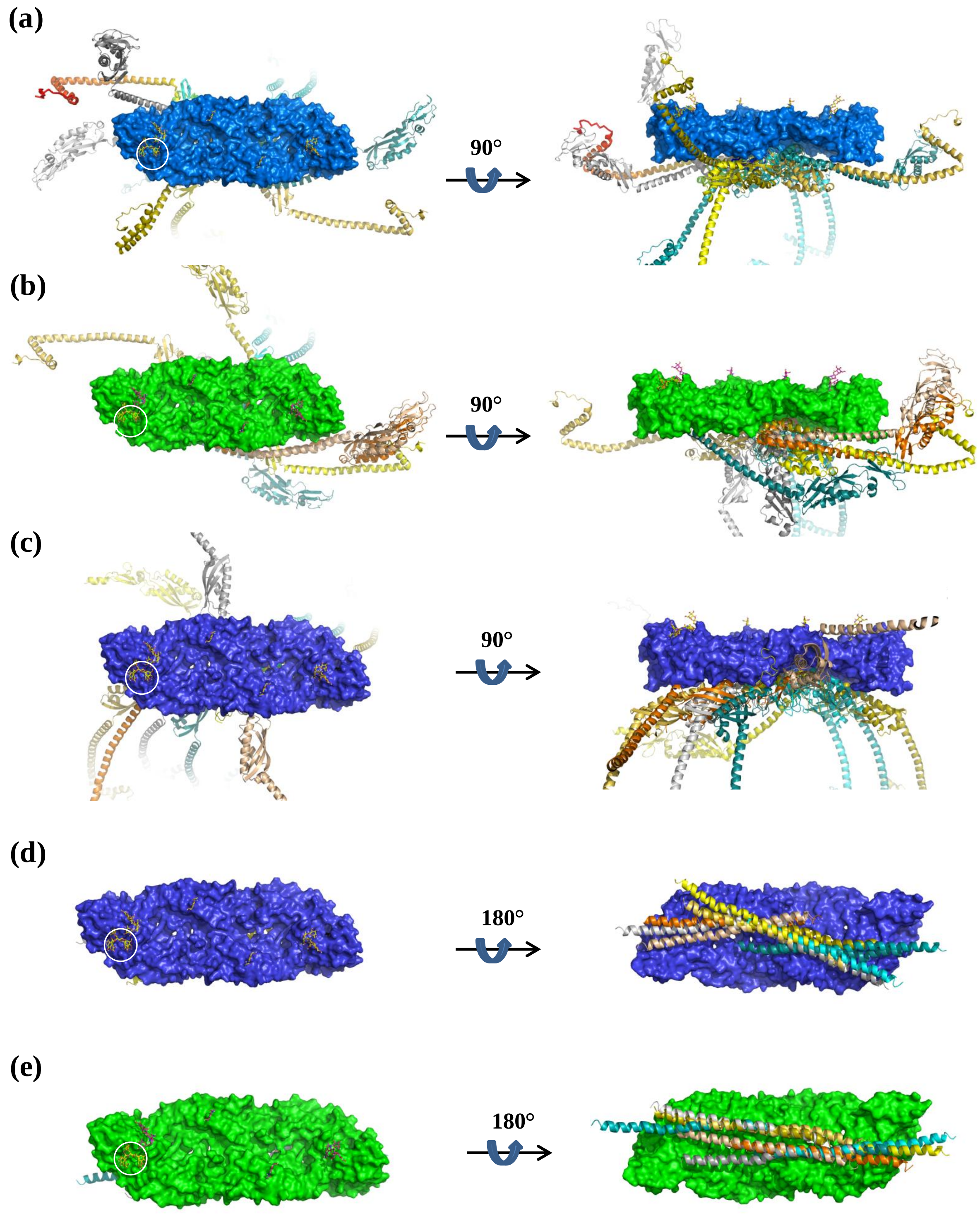

Figure S4: Diagramtic representation of superimposed docked-structures of DENV-2/3 and prohibitin-1/2 proteins and chebulinic acid (CA). The homodimer of DENV-2/3 E protein, prohibitin-1/2, CA and surface glycans of E protien are depicted in the surface, cartoon and sticks representations, respectively. The bound CA, on the surface of the E protein has been encircled for distinction from other surface glycans and for demonstrating the exposed surface of the E protein homodimer. Each diagram represents superimposed structures of the first 10 best docked conformations of DENV E protein dimer and prohibitin proteins and one of the docked structure of E

protein of DENV-2 with CA. (a) DENV-2 E protein and prohibitin-1. (b) DENV-3 E protein and prohibitin-1. (c) DENV-2 E protein and prohibitin-2. (d) DENV-2 E protein and prohibitin-2 coilded coil fragment. (e) DENV-3 E protein and prohibitin-2 coilded coil fragment. None of the docking results showed binding of prohibitins on the exposed surface of DENV-2/3 E homodimer. The structure analysis and the image genetation was performed using the molecular visualization system PyMOL[47]. The docking experiments were performed with modeled structures of prohibitin-1/2 (software SWISS-MODEL[54]), crystal structure of the coliled coil fragment of prohibitin-2 (PDB ID 6IQE) and crystal stucutres of E proteins of DENV-2/3 (PDB ID 1OAN and 1UZG, respectively), using the ClusPro software[56].

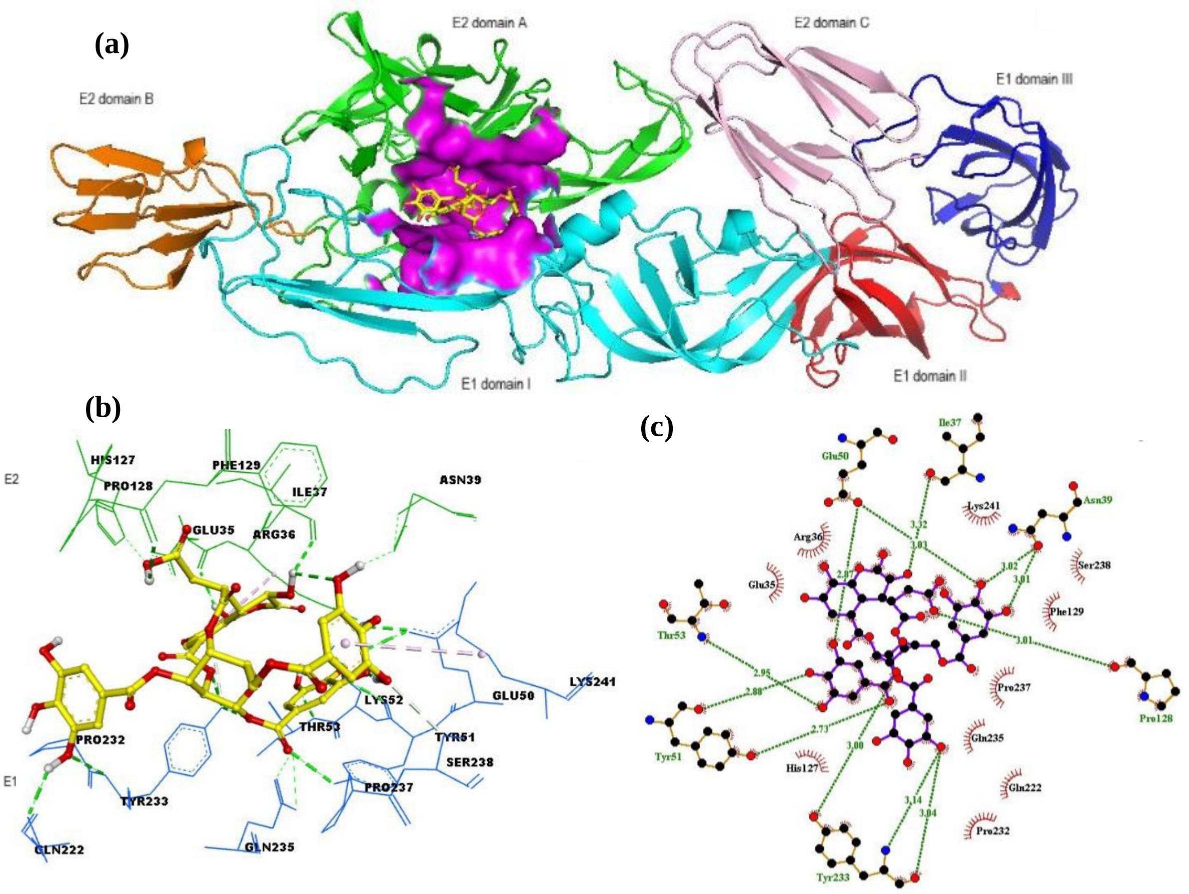

Figure S5: Structure of Chikungunya envelope glycoprotein E1-E2 heterodimer in interaction with chebulinic acid. (a) Ligand (yellow) bound at a major pocket (magenta) at the junction of the heterodimer E1, domain I (cyan), and E2 domain A (green). (b) Depiction of amino acid residues from both E1 and E2 chains participating in the interaction with CA. The green dash lines represent hydrogen bonds, and the pink dash lines represent electrostatic interactions. (c) Schematic diagrams of E2-CA interactions, wherein the hydrogen bonds are depicted in green dashed lines and the hydrophobic contacts are represented in the form of arcs with spokes. Images (a) and (b) were generated using molecular visualization system PyMOL[47], whereas image (c) was produced using the LigPlot software[45, 46].

Table S1: Binding affinity and close contacting residues of (a) DEN-2 E protein, and (b) CHIKV E2 protein, involved in making hydrogen bonds and hydrophobic contacts with chebulinic acid (CA). (c) List of residues of CHIKV E2 protein and prohibitin-1 receptor, involved in making intersubunit contacts at the protein-protein interface.

Docking	Target /Compound	Binding energy (Kcal/mol)	Hydrogen bonds	Minimum distance between H bonds (Å)	Residues forming H bonds	Residues participating in hydrophobic interactions	Residues participating in electrostatic interactions
**(a) DENV E protein /Chebulinic acid**							
Blind Docking	KI β hairpin loop/CA	-8.6	14	2.56	Asn103(A), His244(A), Lys246(A), Lys247(A), Asp249(A), Arg2(B), Ser273(B), Asn276(B)	Ile46(B), Asp154(B), Leu278(B), Asn242(A), Pro243(A),	Asn103(A), Asp154(B), Asn242(A), Asp249(A), Arg2(B), Ser273(B), Asn276(B), Leu278(B),
	Fusion peptide/ CA	-8.3	5	2.741	Ser72(B), Asp98(B), Gly102(B), Ala245(B)	Thr70(B), Glu71(B), Arg99(B), Asn103(B), Ile113(B), Lys246(B), Lys247(B), Arg2(A), Nag1397(A),	Arg2(A), Ser72(B), Lys246(B), Lys247(B), Asn103(B)
Focused docking	EDIII-hinge-ED1/CA	-8.7	14	2.664	Ile357(A), Thr176(A), His158(A), Glu148(A), Glu147(A), Ser145(A), Fuc1399(A), Ser363(A), Tyr299(A), His149(A)	Val365(A), Thr359(A), Val358(A), Lys295(A), Lys160(A), Pro143(A)	Glu147(A), Glu148(A), Lys157(A), Lys160(A), Leu175(A), Thr176(A), Gly177(A), Thr178(A), Lys295(A), Tyr299(A), Thr359(A), Ser363(A),
**(b) CHIKV E2 protein /Chebulinic acid**							
Focused docking	Pose 1	-8.1	7	2.708	Thr12(P), Thr58(P), Thr196(P) His232(P), Lys233(P), Lys234(P),	Asn231(P), Thr230(P), Gln195(P), Gly194(P), Pro75(P), Met74(P), His73(P), Arg68(P), Asp60(P), Arg13(P), Ala11(P), Lys10(P)	-
	Pose 2	-7.4	6	2.743	Ala162(P), Thr160(P), Gly55(P), Phe6(P)	Ala161(P), Met97(P), Trp64(P), Asp63(P), His62(P), Tyr9(P), Val8(P), Asn7(P), Asn5(P), Asp4(P)	-

(c) CHIKV E2 protein /Prohibitin-1		
		Residues of E2 at E2-prohibitin-1 interface
Pose 1	E2	Asp43(P), Trp64(P), Arg80(P), Thr96(P), Arg104(P), Phe141(P), His142(P), Gln158(P), Thr160(P)
	Prohibitin-1	Tyr222(G), Arg226(G), Arg228(G), Thr231(G), Leu233(G), Gln237(G), Ser238(G)
Pose 3	E2	Val8(P), His62(P), Arg80(P), Ile93(P), Thr94(P), Arg144(P), Gln158(P), Ala162(P), Asn263(P), Thr265(P), Cys266(P), Arg267(P),
	Prohibitin-1	Tyr222(G), Arg226(G), Ser227(G), Arg228(G), Asn229(G), Thr231(G), Tyr232(G), Val239(G), Leu241(G)

(A) Chain-A of DENV-2 E protein; (B) Chain-B of DENV-2 E protein, (P) Chain-P of CHIKV E2 protein; (G) Chain-G of prohibitin-1. The hydrogen bond and hydrophobic interactions between (i) DENV-2 E protein and CA and (ii) CHIKV E2 and CA, were analyzed using the LigPlot software[45, 46]; whereas the residues involved in electrostatic interactions between DENV-2 E protein and CA were analyzed using the FLARE software[49].
